# Liraglutide use and evaluation of pancreatic outcomes in a US commercially insured population

**DOI:** 10.1111/dom.13739

**Published:** 2019-05-24

**Authors:** Donnie Funch, Kathleen Mortimer, Najat J. Ziyadeh, John D. Seeger, Ling Li, Heather Norman, Atheline Major‐Pedersen, Heidrun Bosch‐Traberg, Helge Gydesen, David D. Dore

**Affiliations:** ^1^ Optum Epidemiology Boston Massachusetts; ^2^ Global Safety, Novo Nordisk A/S Copenhagen Denmark; ^3^ Global Development, Novo Nordisk A/S Copenhagen Denmark; ^4^ Epidemiology, Novo Nordisk A/S Copenhagen Denmark; ^5^ Department of Health Services, Policy and Practice Brown University School of Public Health Providence Rhode Island

## Abstract

**Aims:**

Both acute pancreatitis (AP) and pancreatic cancer (PC) have been areas of focus for studies of incretin drugs**.** This 5‐year prospective cohort study aimed to quantify possible associations between liraglutide and risk of AP and PC as compared to other antidiabetic drugs (ADs).

**Materials and methods:**

Patients initiating liraglutide or other ADs who were enrolled in a US health plan (2010‐2014) were included. Comparisons of AP and PC incidence rates were made between matched cohorts of liraglutide initiators and initiators of other ADs. Adjudicated AP cases and algorithm‐based PC cases were identified. Propensity score‐matched intention‐to‐treat (ITT) and time‐on‐drug (TOD) analyses were completed using Poisson regression. A latency analysis was performed for PC.

**Results:**

Median follow‐up was 405 days for AP cohorts (9995 liraglutide, 1:1 matched to all comparators) and 503 days for PC cohorts (35 163 liraglutide, 1:1 matched to all comparators*)*. In the primary AP analysis, “current” use of liraglutide was not significantly associated with elevated risk across comparators (all comparators relative risk [RR] = 1.2; 95% confidence interval [CI], 0.6‐2.3). ITT results were similar where, in the primary analysis, no RRs were significantly associated with PC (all comparators RR = 0.7; 95% CI, 0.3‐1.4); latency and TOD analyses did not alter findings. There was no evidence of a dose‐response effect.

**Conclusions:**

Liraglutide was not associated with an increased risk of AP or PC, although risk estimates were more variable for AP, and numbers of cases for both outcomes were limited because of the rarity of outcomes.

## 1 INTRODUCTION

Approximately 30 million people have diabetes in the United States (US),^1^ the majority of which will not meet their therapeutic goals despite treatment with multiple ADs.^2^ Incretin‐based drugs, including glucagon‐like peptide 1 receptor agonists (GLP‐1 RAs), are an important addition to the therapeutic options available for treatment of type 2 diabetes (T2D).^3^ This class of drugs is associated with a reduction in glycated haemoglobin (HbA1c), weight loss and minimal risk of hypoglycaemia.^4^, ^5^, ^6^, ^7^ A reduced risk of cardiovascular events was also seen for some drugs in this class.^8^, ^9^

After introduction of the first GLP‐1 RA, exenatide twice‐daily, questions were raised about an increased risk of acute pancreatitis (AP) and pancreatic cancer (PC). The US Food and Drug Administration (FDA) and European Medicines Agency reviewed the totality of existing data but, to date, a final conclusion has not been rendered regarding a causal association between GLP‐1 RA therapies and either AP or PC; therefore, they remain safety risks for these drugs. Both agencies called for more research in this area.^10^, ^11^ Thus, this post‐marketing regulatory requirement supplements the growing body of studies utilizing real world data and methods to address concerns about confounding and bias in observational studies. A previously published brief report provided early results^12^; this manuscript presents the final analyses.

Several studies using randomized clinical trial (RCT) methods or observational methods have already been published concerning this topic. Glycaemic control trials of liraglutide,^13^ which belongs to the GLP‐1 RA class, revealed more cases of AP in liraglutide arms as compared with control arms, although this was not confirmed in the Liraglutide Effect and Action in Diabetes: Evaluation of Cardiovascular Outcome Results (LEADER) trial.^14^ While RCTs have been used in a number of evaluations, data from observational studies provide an important source of supplemental evidence.^15^ Non‐clinical data concerning the effects of GLP‐1 mimetic drugs do not support a mechanism of action affecting the exocrine pancreas.^16^ Numerous observational studies, as well as RCTs, have examined the risk of AP or PC in relation to GLP‐1 RA and other incretin therapies (dipeptidyl peptidase‐4 inhibitor [DPP‐4*i*]), with mixed findings.^17^ Recent meta‐analyses, however, have provided increasing support that pancreatitis and PC are not a major concern in populations treated with incretin‐based therapies.^15^, ^18^, ^19^ This study aimed to estimate the risks of AP and PC in cohorts of patients initiating liraglutide as compared to initiators of other non‐insulin ADs.

## 2 MATERIALS AND METHODS

### 2.1 Data source

This prospective cohort study was conducted using the Optum Research Database (ORD), which includes a large, geographically diverse population of commercial health insurance enrollees. The ORD contains enrollment data, medical claims and pharmacy claims; enrollment in 2014 included approximately 13.9 million individuals with both medical and pharmacy benefits and comprises approximately 4% of the US population. This study was approved by the New England Institutional Review Board and Privacy Board.

### 2.2 Study design and study data

A new‐user, active‐comparator design was used for this study.^20^ Patients were adult initiators of liraglutide or another non‐insulin AD who had been continuously enrolled for at least 6 months prior to initiation of a study drug (baseline period), with no dispensing of that drug or drug class during baseline. The accrual period was 1 February 2010 to 31 December 2013 for the AP cohort, and recruitment for the PC cohort continued through 30 November 2014. For comparison to liraglutide, cohorts were created for exenatide, metformin, pioglitazone, a sulfonylurea (SU) (glyburide, glipizide or glimepiride) or a DPP‐4*i* (sitagliptin, saxagliptin or linagliptin) and for three summary comparators: all comparators combined, all comparators excluding exenatide, to address potential GLP‐1 RA effect, and all comparators excluding exenatide and DPP‐4i, to address potential incretin effect.

Follow‐up began on the day after first observed dispensing of a study drug (index date) and continued until disenrollment, censoring or end of study period (31 March 2014 for AP, 31 December 2014 for PC), whichever took place first. The AP cohorts were restricted to the sub‐population for whom medical records could be requested to confirm AP outcome and patients with a diagnosis of AP or chronic pancreatitis (ICD‐9577.1) at baseline were excluded. Patients with a PC diagnosis during baseline were excluded from both cohorts.

### 2.3 Propensity Score (PS) matching

Logistic regression modeled the PS as the predicted probability of initiating liraglutide vs comparison treatment, conditional on covariates.^21^ A number of covariates were included in the PS to address confounding,^22^ with some forced into the model, including age, region, health care utilization metrics, number of unique ADs, diabetes severity,^23^ AD use and outcome‐specific risk factors. For AP cohorts, additional covariates included abdominal pain, cholelithiasis, cholecystectomy, pancreatic disease, overweight and smoking/alcohol use.^24^ For PC cohorts, covariates for pancreatitis, other pancreatic disease, smoking/alcohol use and race were included. To account for changes in prescribing patterns and availability of ADs on the market, PS estimation and matching were performed within calendar periods for each comparator.^25^ For the first three years, cohorts were created by calendar quarter and annually thereafter for the remaining recruitment time. Separately for AP and PC analyses, the eight comparator cohorts were matched 1:1 to liraglutide initiators within each period using a greedy matching algorithm.^26^

Many patients initiated more than one study drug. Subsequent initiators who did not match during one recruitment time block, but who initiated any study drug during a later block, were again eligible for matching with PS, re‐estimated using the baseline period prior to that initiation. Patients could enter into multiple matched cohorts over the study period but were allowed to match into a drug cohort pair only once.

### 2.4 Outcome definitions

Potential AP cases were initially identified by ICD‐9 code 577.0, which does not include chronic pancreatitis, cysts or pseudocysts of the pancreas, or other diseases of the pancreas. Because the positive predictive value (PPV) for AP is typically low,^27^ medical charts were reviewed by a gastroenterologist. The algorithm for confirming AP as “definite” or “probable” included:

1. Report of abdominal pain **and;**
2. Lipase levels ≥3× upper limit normal (ULN) or 300 units per liter (U/L) (if normal range not noted) **or** amylase levels ≥5× ULN or 1000 U/L (if normal range not noted) **and/or;**
3. Imaging diagnostic for, or suggestive of, AP.^28^

Definite cases (three of the three criteria) and probable cases (criterion 1 and criterion 2 or 3) were considered confirmed cases. Final analyses included cases of AP that were confirmed through this medical chart review/adjudication.

PC cases were identified, employing a validated algorithm (PPV of 0.88 [95% CI 0.62‐0.98])^29^ which required all of the following criteria:

- Claim with an inpatient or outpatient diagnosis of PC (ICD‐9157.xx)
- No diagnosis of benign pancreatic neoplasm within 60 days after PC diagnosis
- Procedures indicating pancreatic surgery, chemotherapy or radiation therapy within 180 days after PC diagnosis
- No diagnosis of other cancers (ICD‐9150.xx‐156.xx, 158.xx, 159.xx, 162.xx, 165.xx, 171.5, 188.xx, 195.2) within 60 days before or after PC diagnosis

### 2.5 Analyses

Baseline covariates and distribution of time‐to‐event for AP and PC were summarized for liraglutide initiators and all comparators. Analytic methods separately quantified risks associated with drug initiation, recency of use and cumulative exposure. ITT analyses quantified the risk associated with initiating treatment, attributing follow‐up time to drug used at matched cohort entry. Incidence rates (IR per 100 000 person‐years [PYs]) were estimated as the number of cases divided by PYs at‐risk. Poisson regression was used to compute estimated incidence rate ratios (RRs) and 95% CIs. Generalized estimating equations (GEE) were used to account for the paired nature of the matched cohorts.^30^ For PC, a sensitivity analysis was performed, excluding the first year of follow‐up to avoid inclusion of prevalent cases and to account for expected latency of PC.

For each outcome, TOD analyses evaluated the effect of treatment within ‘current’, ‘recent’, and ‘past’ categories. “Current” use included first day of follow‐up through end of days supplied, with an additional 31 days to account for medication non‐adherence. Refill dispensings that were observed during these 31 days resulted in continuous “current” use time. “Recent” use began at the end of “current” time and continued for another 31 days. Subsequent person‐time was categorized as “past” use, which persisted unless the patient re‐initiated the same treatment, thereby re‐entering “current” use. Poisson regression was used to estimate the RRs and 95% CIs for “current,” “recent” and “past” use of liraglutide vs the corresponding comparator category, adjusted by the logit of the PS to address confounding.

Another TOD analysis addressed potential for a dose‐response relationship through calculation of cumulative time on liraglutide. Cumulative time exposed and unexposed to liraglutide during follow‐up was quantified for PC, and IR was determined within each exposure type. Estimates of RR were made using Poisson regression within categories of cumulative time (short [<6 months], moderate [6‐18 months] and long [>8 months]) relative to all “liraglutide unexposed” time, adjusted for the logit of the PS. The TOD analysis was the primary analysis for AP, with focus on “current” time, given the acute nature of AP, while the ITT analysis was the primary analysis for PC.

## 3 RESULTS

### 3.1 Acute pancreatitis

Among first observed initiations for each patient, 8499 initiated liraglutide and 100 161 initiated comparators. A total of 9995 liraglutide initiations, including first and subsequent initiations, were 1:1 matched to all comparator initiations. Selected baseline covariate distributions for the liraglutide:all comparators pair before and after matching for the AP analysis are displayed in Table 1. The matched pairs were well balanced. Distributions for all covariates are available in Appendix S1 (Table S1a) in File S1.

**Table 1:** Baseline descriptive characteristics of all initiators, pre‐ and post‐matching,^a^ Acute Pancreatitis CohortOptum Research Database

	First initiation at cohort entry				All matched initiators (initial and subsequent initiations)^b^			
	Liraglutide N = 8499		All comparators N = 100 161		Liraglutide N = 9995		All comparators N = 9995	
Description	N	%	N	%	N	%	N	%
**Demographic**								
*Age (mean [median], IQR)*	52(53)	46.0 to 59.0	51(53)	44.0 to 59.0	52(53)	46.0 to 59.0	52(53)	46.0 to 59.0
18 to 39	984	11.6	16 676	16.6	1137	11.4	1185	11.9
40 to 49	2183	25.7	23 017	23.0	2541	25.4	2540	25.4
50 to 59	3334	39.2	36 091	36.0	3931	39.3	3944	39.5
60 to 64	1459	17.2	16 494	16.5	1713	17.1	1696	17.0
65+	539	6.3	7883	7.9	673	6.7	630	6.3
*Gender*								
Female	4484	52.8	47 997	47.9	5247	52.5	5287	52.9
*Race*								
Black	928	10.9	13 109	13.1	1125	11.3	1285	12.9
**Healthcare utilization**								
*Total cost ($) (median, IQR)*	3675	2052 to 6509	1835	762 to 4305	3578	2024 to 6394	3487	1980 to 6414
*Number of drugs*								
0 to 4	830	9.8	30 998	30.9	943	9.4	1004	10.0
5 to 7	1990	23.4	31 420	31.4	2380	23.8	2348	23.5
8 to 10	2337	27.5	19 895	19.9	2791	27.9	2786	27.9
11+	3342	39.3	17 848	17.8	3881	38.8	3857	38.6
*Any Hospitalization*	390	4.6	6800	6.8	476	4.8	469	4.7
**Baseline Conditions**								
*Overweight*	1670	19.6	12 544	12.5	1941	19.4	1912	19.1
*DCSI Score* c								
0	6072	71.4	79 385	79.3	7159	71.6	715	71.2
1	1320	15.5	10 598	10.6	1526	15.3	1571	15.7
2	696	8.2	6532	6.5	820	8.2	807	8.1
3+	411	4.8	3646	3.6	490	4.9	502	5.0
**History of Baseline Diseases/Procedures**								
*Type 2 diagnosis*	7231	85.1	70 319	70.2	8581	85.9	8604	86.1
*No diabetes diagnosis*	1116	13.1	29 029	29.0	1262	12.6	1266	12.7
*Pancreatic disease*	1	0.0	31	0.0	2	0.0	4	0.0
*Abdominal pain*	714	8.4	8746	8.7	872	8.7	837	8.4
*Cholelithiasis*	30	0.4	513	0.5	41	0.4	46	0.5
*Status post cholecystectomy*	29	0.3	335	0.3	40	0.4	33	0.3
*Abdominal ultrasound*	267	3.1	2948	2.9	324	3.2	312	3.1
*Diabetes‐related nephropathy*	358	4.2	1861	1.9	397	4.0	361	3.6
*Diabetes‐related neuropathy*	691	8.1	3594	3.6	766	7.7	764	7.6
*Diabetes‐related retinopathy*	273	3.2	1600	1.6	307	3.1	289	2.9
*Hyperlipidaemia*	5739	67.5	52 561	52.5	6758	67.6	6762	67.7
**Baseline Drug Use**								
*Cholesterol‐lowering drugs*	4745	55.8	43 218	43.1	5573	55.8	5561	55.6
*Insulin use*	2309	27.2	8704	8.7	2533	25.3	2417	24.2
*Number of unique antidiabetic drugs* d								
0	2342	27.6	65 842	65.7	2517	25.2	2529	25.3
1	2950	34.7	23 984	23.9	3537	35.4	3593	35.9
2+	3207	37.7	10 335	10.3	3941	39.4	3873	38.7

*Note:* Initiation period: 1 February 2010 to 31 December 2013; Follow‐up through 31 March 2014.

a Patients were allowed to initiate multiple times during the study period and to match only once into each drug cohort pair.

b Not including insulin.

c Patients who do not match based on their first initiated drug were eligible to be matched based on subsequent drug initiations.

d Diabetes Complications Severity Index.

Overall median follow‐up time was 405 days (interquartile range [IQR] 181‐747 days), ranging from 335 days (SUs) to 469 days (pioglitazone). Within each matched set, median lengths of follow‐up were similar.

Charts were requested for 350 patients with an AP diagnosis. Overall, 180 AP cases were confirmed through adjudication; 15 were dropped for administrative reasons or baseline exclusions, leaving 165. More details on the adjudication process are available in Appendix S2 in File S1.

Median time from matched cohort entry until AP diagnosis was 421 days (IQR,248‐611) for liraglutide compared with 286 days (IQR, 127‐565) for all comparators. Cases in the liraglutide cohorts experienced consistently longer times to diagnosis. Distributions of time to AP diagnosis are available for all pairs in Appendix S3 in File S1.

**Figure 1:**
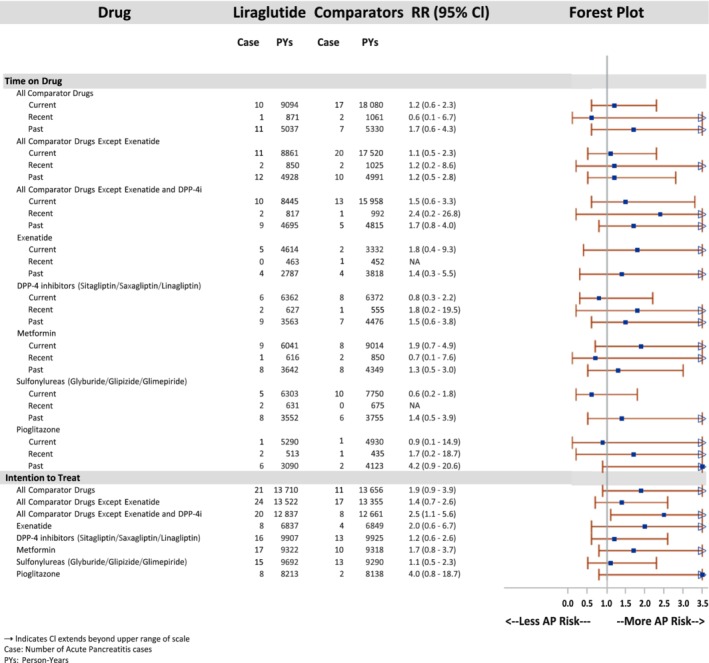
Propensity‐score‐matched time on drug and intention‐to‐treat analyses for acute pancreatitis

The results of AP analyses are presented in Figure 1. In the TOD analysis, RRs for “current” use ranged from 0.6 (95% CI, 0.2‐1.8, sulfonylurea) to 1.9 (95% CI, 0.7‐4.9, metformin). RRs varied substantially for “recent” use; however, these RRs were based on small numbers of cases and limited PYs, and thus could not be calculated for two comparisons. In general, RRs for “past” use were higher than estimates of “current” and “recent” use.

In the ITT analyses, RRs ranged from 1.1 (95% CI, 0.5‐2.3, sulfonylurea) to 4.0 (95% CI, 0.8‐18.7, pioglitazone), with one statistically significant elevated risk (RR 2.5 [95% CI, 1.1‐5.6, all comparators except exenatide and DPP‐4is]).

### 3.2 Pancreatic cancer

Among first observed initiations for each patient, 27 283 were liraglutide initiations and 362 539 were initiations of any comparator. A total of 35 163 liraglutide initiations, including first and subsequent, were matched to all comparators initiations. Distributions of select baseline covariates pre‐ and post‐matching for the liraglutide:all comparators pair are displayed in Table 2 (see also Appendix S1, Table S1b in File S1). Similar covariate balance was found for all other pairs. Median length of follow‐up was 503 days (IQR, 216‐966). Length of follow‐up varied across matched drug cohorts, but was similar within.

**Table 2:** Baseline descriptive characteristics of all initiators, pre‐ and post‐matching^a^, Acute Pancreatitis Cohort, Optum Research Database

	First initiation at cohort entry				All matched initiators (initial and subsequent initiations)^b^			
	Liraglutide N = 27 283		All comparators N = 362 539		Liraglutide N = 35 163		All comparators N = 35 163	
Description	N	%	N	%	N	%	N	%
**Demographic**								
*Age (mean [median], IQR)*	52 (53)	45.0 to 59.0	52 (53)	44.0 to 61.0	52 (53)	46.0–60.0	52 (53)	46.0–59.0
18 to 39	3181	11.7	60 908	16.8	3970	11.3	3974	11.3
40 to 49	6898	25.3	78 217	21.6	8848	25.2	8983	25.5
50 to 59	10 549	38.7	120 024	to	13 516	38.4	13 540	38.5
60 to 64	4594	16.8	58 895	16.2	5999	17.1	5950	16.9
65+	2061	7.6	44 495	12.3	2830	8.0	2716	7.7
*Gender*								
Female	14 644	53.7	178 007	49.1	18 712	53.2	18 699	53.2
Male	12 639	46.3	184 532	50.9	16 451	46.8	16 464	46.8
*Race*								
Black	3283	12.0	50 420	13.9	4282	12.2	4971	14.1
**Healthcare Utilization**								
*Total Cost ($) (median, IQR)*	3947	2183 to 7050	1991	832 to 4641	3920	2185 to 7011	3705	2166 to 6843
*Any ER visit*	4963	18.2	71 340	19.7	6484	18.4	6450	18.3
*Any hospitalization*	1252	4.6	26 481	7.3	1716	4.9	1664	4.7
**Baseline conditions**								
*Overweight*	5786	21.2	48 929	13.5	7478	21.3	7494	21.3
*DCSI Score* c								
0	19 413	71.2	282 762	78.0	24 965	71.0	24 593	69.9
1	4207	15.4	38 295	10.6	5381	15.3	5624	16.0
2	2237	8.2	25 788	7.1	2953	8.4	3048	8.7
3+	1426	5.2	15 694	4.3	1864	5.3	1898	5.4
**History of baseline diseases/procedures**								
*Type 2 diabetes*	23 239	85.2	254 223	70.1	30 505	86.8	30 671	87.2
*No diabetes diagnosis*	3558	13.0	105 623	29.1	4129	11.7	4052	11.5
*Benign thyroid disease*	3702	13.6	32 066	8.8	4629	13.2	4579	13.0
*Goiters/nodules*	960	3.5	6901	1.9	1199	3.4	1180	3.4
*Pancreatic disease*	62	0.2	1658	0.5	79	0.2	131	0.4
*Acute pancreatitis*	46	0.2	1291	0.4	57	0.2	102	0.3
*Chronic pancreatitis*	15	0.1	401	0.1	21	0.1	34	0.1
*Diabetes‐related nephropathy*	1162	4.3	7570	2.1	1510	4.3	1421	4.0
*Diabetes‐related neuropathy*	2177	8.0	13 634	3.8	2772	7.9	2762	7.9
*Diabetes‐related retinopathy*	865	3.2	5672	1.6	1084	3.1	1065	3.0
*Hyperlipidaemia*	18 336	67.2	189 873	52.4	23 858	67.8	23 698	67.4
**Baseline drug dispensings**								
*Cholesterol‐lowering drugs*	15 273	56.0	160 058	44.1	19 825	56.4	19 595	55.7
*Insulin use*	7762	28.4	32 370	8.9	9387	26.7	9266	26.4
*Number of unique antidiabetic drugs* d								
0	7747	28.4	240 394	66.3	8682	24.7	8552	24.3
1	9676	35.5	86 422	23.8	12 677	36.1	12 899	36.7
2+	9860	36.1	35 723	9.9	13, 04	39.3	13 712	39.0

*Note:* Initiation period: 1 February 2010 to 31 December 2013; Follow‐up through 31 March 2014.

a Patients were allowed to initiate multiple times during the study period and to match only once into each drug cohort pair.

b Patients who do not match based on their first initiated drug were eligible to be matched based on subsequent drug initiations.

c Diabetes Complications Severity Index.

d Not including insulin.

**Figure 2:**
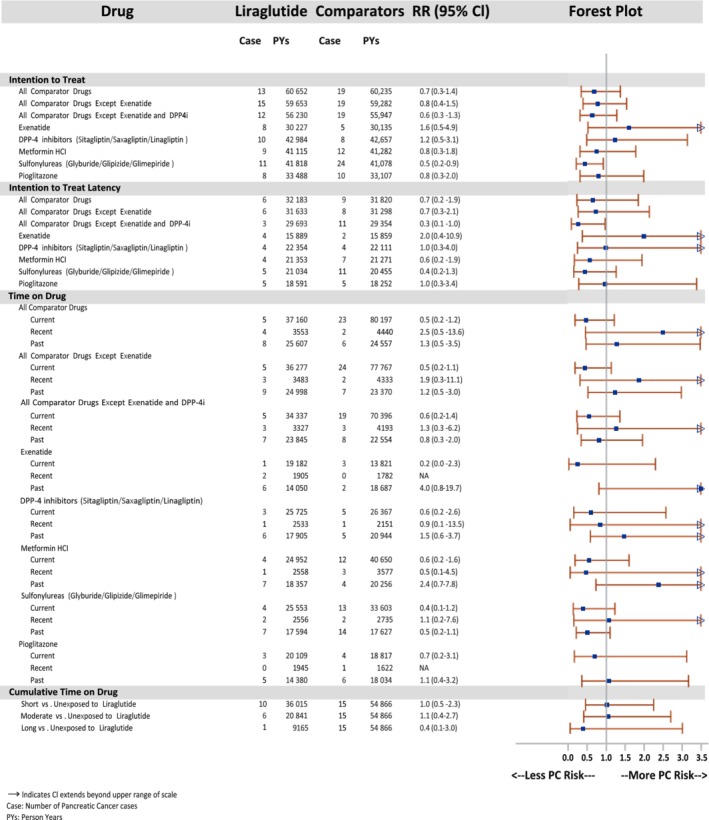
**Propensity‐score matched intention‐to‐treat and time‐on‐drug analyses for pancreatic cancer**

A total of 240 claims‐based cases were identified, with 10 among liraglutide initiators. Median days to PC diagnosis were 369 (IQR, 226‐1099, liraglutide) and 318 (IQR, 161‐627, all comparators) (see Appendix S3 in File S1 for time to PC diagnosis). Results of PC analyses are displayed in Figure 2.

In the primary analysis (ITT), IRs were 21.4/100 000 PYs (liraglutide) vs 31.5/100 000 PYs (*all* comparators). RRs ranged from 0.5 (95% CI, 0.2‐0.9, sulfonylurea) to 1.6 (95% CI, 0.5‐4.9, exenatide). Most RRs were similar or lower when a one‐year latency period was taken into account (data not shown).

In the TOD analysis, RRs for “current” use ranged from 0.2 (95% CI, 0.0‐2.3, exenatide) to 0.7 (95% CI, 0.2‐3.1, pioglitazone). RRs were higher for “recent” use, but these estimates were based on small numbers of cases and limited PYs. The two highest RRs were in “past” use ([RR, 4.0; 95% CI, 0.8‐19, exenatide] and [RR, 2.4; 95% CI, 0.7‐7.8, metformin]).

In the cumulative TOD analysis, adjusted RRs were 1.0 (95% CI, 0.5‐2.3, short), 1.0 (95% CI, 0.4‐2.7, moderate) and 0.4 (95% CI, 0.1‐3.0, long).

## 4 DISCUSSION

This five‐year prospective cohort study included several drug cohorts sourced from administrative claims, supplemented with medical record review. Across therapies with different mechanisms of action, the data do not suggest an association between liraglutide use and increased risk of AP or PC.

In the AP TOD analysis, RRs often exceeded 1.0; however, no findings excluded the null. The estimated RR was not greater than 1.0 for contrasts within “current” exposure. The question of whether patients with early symptoms of AP may have switched medications and discontinued use of liraglutide more rapidly than comparators, which would result in more cases being attributed to the “start of past use” liraglutide category relative to comparators, was investigated via sensitivity analysis. Mean time between the beginning of “start of past use” and date of AP cases was longer for liraglutide (265 days [median, 120 days]) than for all comparators (239 days [median, 190 days]), a finding that does not indicate that liraglutide patients were more likely to have discontinued or switched medications prior to diagnosis of AP.

In the ITT analyses, only the CI for all comparators except exenatide and DPP‐4i excluded the null. Another sensitivity analysis that evaluated the potential selection bias that could result from preferential discontinuation following AP symptoms was completed for each of the eight matched AP cohorts. Baseline characteristics of patients who discontinued the study drug early during follow‐up (≤median) and those of patients who discontinued use later during follow‐up were compared. Occasional differences existed; for example, among patients who discontinued early, liraglutide patients were slightly older and more likely to be black than were DPP‐4*i* patients. However, no consistent patterns of patient differences were observed overall, suggesting balanced characteristics in the cohorts over time, regardless of timing in relation to discontinuation.

Several observational studies, including early results from this study, found no significant association between GLP‐1 RAs (generally exenatide) and AP.^12^, ^31^, ^32^, ^33^, ^34^ Garg and colleagues compared exenatide and sitagliptin initiators with a diabetic control group with participants initiating other ADs. The adjusted hazard ratio (aHR) for exenatide was 0.9 (95% CI, 0.6‐1.5). Another cohort study that included liraglutide found no elevation in risk for “current” use of GLP‐1 RAs (aHR, 1.11 [95% CI, 0.54‐2.26]) compared with elevated risk for “current” use of DPP‐4*i* therapies (aHR, 1.59 [95% CI, 1.05‐2.40]).^35^ Elashoff et al. reported significant elevations for both GLP‐1 and DPP‐4*i* therapies,^36^ but this study was based on the FDA Adverse Event Reporting System database, which has known limitations.^37^ One case‐control study of incretins, exenatide and sitagliptin found elevations in risk for “current” use (adjusted odds ratio [aOR], 2.24 [95% CI 1.36‐3.68]) and “recent” use (aOR, 2.01 [95% CI, 1.37‐3.18]) but did not quantify risk with exenatide and with sitagliptin separately.^38^

A meta‐analysis of RCTs of GLP‐1 therapies and pancreatitis found no significant increase in risk across a range of comparators (Mantel‐Haenszel [M‐H] OR, 0.93 [95% CI, 0.65‐1.34]).^19^ A sub‐group analysis including only trials with adjudication of AP had similar results (M‐H OR, 0.87 [95% CI, −0.53‐1.44]). Another meta‐analysis of three RCTs of GLP‐1 therapies looking at AP as a predefined and independently adjudicated adverse event also found no elevated risk in T2D patients (Peto OR, 0.745 [95% CI, 0.47‐1.17]).^39^ This result is in contrast to that of two similar meta‐analyses of DPP‐4*i* RCT that found significant increases in AP risk in the treatment group vs controls. These studies were based on the same three RCTs with large patient populations, adjudicated cases of AP and median follow‐up periods ranging from 1.6 to 3.0 years.^40^, ^41^ As expected, the results were nearly identical and suggested an approximately 80% increase in risk of AP with DPP‐4*i* use (OR, 1.79 [95% CI, 1.13‐2.82]^40^ and OR, 1.82 [95% CI, 1.17‐2.82]^41^). A recent trial among patients with pre‐diabetes using higher doses of liraglutide (3.0 mg) for weight loss also reported a higher proportion of AP diagnoses among the liraglutide group (0.7%; 12 cases) vs those who received placebo (0.3%; two cases).^42^ Overall, however, there appears to be increasing support for the absence of association between GLP‐1 RA therapies and AP.^15^

In PC analyses, the majority of ITT RRs were below 1.0; the only statistically significant finding was a lower risk with liraglutide use, relative to SUs. No dose‐response effect was observed in the cumulative use analyses, consistent with other observational studies of the GLP‐1 RA class, with the exception of that by Elashoff et al, with the limitations already noted.^36^, ^43^, ^44^, ^45^ In a retrospective cohort study with over three years of follow‐up, Knapen et al conducted a TOD analysis and did not find a significantly elevated risk with “current” use of GLP‐1 RAs (aHR, 1.43 [95% CI, 0.96‐2.13]) or DPP‐4*i*s (aHR, 1.18 [95% CI, 0.52‐2.69]) independently or combined (aHR, 1.36 [95% CI, 0.94‐1.96]).^43^ An exenatide cohort study found no elevated risk when compared with metformin or glyburide (RR 0.8 [95% CI 0.5‐1.6]) in 1 year of follow‐up.^44^ A large international cohort study also found no significant elevation if the first year of follow‐up was discounted (aHR, 1.13 [95% CI, 0.38‐3.38]).^45^ The follow‐up period ranged between 1.3 and 2.8 years across sites, not including the one discounted year. A recent summary of RCTs of GLP‐1 RAs revealed no elevation in PC risk (Peto OR, 0.77 [95% CI, 0.42‐1.42]) comparing GLP‐1 RAs with either placebo or control.^46^ In addition, recent meta‐analyses have provided additional evidence that PC is not a major concern in populations treated with incretin‐based therapies.^19^

The strengths of this study include the use of a large administrative claims database, supplemented with a medical record review. Case ascertainment included medical record adjudication (AP) and a validated algorithm (PC).^29^ Numerous ADs were evaluated, and cohorts were balanced in a wide range of potential confounders. An important limitation of studies sourced from health insurance claims data is that some important potential confounders (eg, BMI, smoking/alcohol use) may not be well characterized; however, PS matching has been shown to improve balance in unmeasured characteristics, including clinical parameters, as many measured covariates are proxies for unmeasured information.^22^, ^47^ Duration of follow‐up may be limited in the ORD as the result of individuals changing health insurance plans; thus, the ability to assess the effect of study drugs on outcomes that occur more than 1 to 2 years after initiation is limited, which may affect the interpretation of results concerning those outcomes with longer induction periods (ie, PC).

Each cohort, including the all comparator cohorts, was created separately and was PS‐matched with the liraglutide cohort, independently, within refined calendar periods to capture changes in US market share. Three all comparator cohorts were used, so that, in addition to including a matched comparison with all study comparators, one comparison involves non‐GLP1‐RA drug classes and another involves non‐incretins.

Multiple analytic approaches were used to balance the strengths and weaknesses. Because AP is an acute event, TOD was the primary analysis, with a focus on “current” use. In contrast, longer‐term exposure was important to assess the risk of PC; thus, ITT was the primary analysis, including latency analysis to reduce potential protopathic bias.

Medical record confirmation was required for AP cases, restricting the sample size to patients in plans that allow access to medical records (~30% of eligible patients). Despite this restriction, the number of AP cases is comparable to that of many other individual RCTs and observational studies with chart‐confirmed outcomes.^19^, ^39^, ^48^ The number of PC cases was small also because of the rarity of the outcome. PC cases were identified using an algorithm, possibly introducing misclassification; however, severity of PC, rapid time course once diagnosed, and use of an algorithm developed in the source database contribute to the accuracy of case identification. The number of cases was similar to that of those in studies included in pooled RCT analyses of GLP‐1 drugs and in several other observational studies focused on PC.^19^, ^43^, ^44^, ^46^

Observational studies assess associations under “real‐world” conditions; however, for a disease such as diabetes with numerous treatment options, these conditions complicate AD studies. Many patients have a history of AD use, and even restricted definitions of initiation may not fully capture earlier drug use. Concurrent or add‐on drugs in other classes are difficult to quantify. Patients switch/add therapies if side effects occur or if diabetes is not well‐managed. Baseline drug use was included in the PS; however, changes during follow‐up are not accounted for with these analytic methods. As a result, it is difficult to attribute causality among patients using multiple drugs or undergoing multiple therapies.

Results should be interpreted with the understanding that comparators may not be reflective of initiators of those individual drugs in general, particularly for first‐line therapies, e.g., the comparison is made between liraglutide initiators and metformin initiators with baseline characteristics similar to liraglutide initiators, and not a comparison to all types of metformin initiators.

Many measures and comparators were used to assess the association between liraglutide and pancreatic outcomes. AP analyses demonstrate no consistent pattern of increased risk with liraglutide during any period of use, including the primary analysis of “current” use. There was no support for an increased risk of PC with liraglutide use, a finding that is consistent with many other studies.

In conclusion, based on observational data in a commercially insured population, liraglutide use was not associated with increased risk of AP or PC. Although high specificity of identified cases was achieved, concerns remain regarding the limited numbers because of the rarity of outcomes, as well as confounding by unmeasured factors and the question of whether the findings are generalizable to other populations.

## CONFLICT OF INTEREST

The study was supported through a research contract between Optum and Novo Nordisk A/S, who is the Marketing Authorization Holder of liraglutide. N.J.Z., J.D.S., H.N. and D.D.D. are employed by Optum and hold stock/stock options in the parent company of Optum (United HealthGroup, Inc.). D.F., K.M. and L.L. were employed by Optum at the time of study conduct. A.M.‐P., H.B.‐T. and H.G. are employees and stockholders of Novo Nordisk A/S.

## AUTHOR CONTRIBUTIONS

D.D.D. had full access to all data in the study and takes responsibility for the integrity of the data and the accuracy of the data analysis. Because this work was undertaken to meet a regulatory request, both the FDA and Novo Nordisk reviewed and commented on the protocol and statistical analysis plan underlying this work. The final study design and analytic plan required FDA approval. D.D.D., D.F. and K.M. were responsible for study concept and design. L.L. and H.N. were responsible for acquisition of data. D.D.D., D.F., K.M., L.L. and H.N. were responsible for analysis and interpretation of data. D.F. and N.J.Z. were responsible for drafting the manuscript. D.D.D., K.M., H.G., A.M.‐P., H.B.‐T., N.J.Z. and J.D.S. were responsible for critical revision of the manuscript for important intellectual content.

## Supporting information


**File S1.** Supplementary Appendices.
