# Supplementary material for: Liraglutide use and evaluation of pancreatic outcomes in a US commercially insured population
Source: Diabetes Obes Metab. 2019 May 24;21(8):1837–48. doi: 10.1111/dom.13739 (PMC6772183; doi:10.1111/dom.13739)
Supplement: Supplementary file 1 — File S1. Supplementary Appendices. [file DOM-21-1837-s001.docx]

**Supplementary Appendices**

**Supplemental Appendix 1. Baseline Covariates before and after matching**

| **Table S1a. Baseline Descriptive Characteristics for All Initiators, Pre- and Post-Matching^1^, Acute Pancreatitis Cohort Optum Research Database Initiation period: February 1, 2010 - December 31, 2013**  **Follow-Up Through March 31, 2014** | | | | | | | | |
| --- | --- | --- | --- | --- | --- | --- | --- | --- |
| **Description** | **First Initiation at Cohort Entry** | | | | **All Matched Initiators  (Initial and Subsequent Initiations)^2^** | | | |
|  | **Liraglutide N=8,499** | | **All Comparators N=100,161** | | **Liraglutide N=9,995** | | **All Comparators N=9,995** | |
|  | **N** | **%** | **N** | **%** | **N** | **%** | **N** | **%** |
| **Demographic** |  |  |  |  |  |  |  |  |
| *Age (mean (median), IQR)* | 52(53) | 46.0-59.0 | 51(53) | 44.0-59.0 | 52(53) | 46.0-59.0 | 52(53) | 46.0-59.0 |
| *Age (median, IQR)* | 53 | 46.0-59.0 | 53 | 44.0-59.0 | 53 | 46.0-59.0 | 53 | 46.0-59.0 |
| 18-39 | 984 | 11.6 | 16,676 | 16.6 | 1,137 | 11.4 | 1,185 | 11.9 |
| 40-49 | 2,183 | 25.7 | 23,017 | 23.0 | 2,541 | 25.4 | 2,540 | 25.4 |
| 50-59 | 3,334 | 39.2 | 36,091 | 36.0 | 3,931 | 39.3 | 3,944 | 39.5 |
| 60-64 | 1,459 | 17.2 | 16,494 | 16.5 | 1,713 | 17.1 | 1,696 | 17.0 |
| 65+ | 539 | 6.3 | 7,883 | 7.9 | 673 | 6.7 | 630 | 6.3 |
| *Gender* |  |  |  |  |  |  |  |  |
| Female | 4,484 | 52.8 | 47,997 | 47.9 | 5,247 | 52.5 | 5,287 | 52.9 |
| Male | 4,015 | 47.2 | 52,164 | 52.1 | 4,748 | 47.5 | 4,708 | 47.1 |
| *Race* |  |  |  |  |  |  |  |  |
| Black | 928 | 10.9 | 13,109 | 13.1 | 1,125 | 11.3 | 1,285 | 12.9 |
| *Region* |  |  |  |  |  |  |  |  |
| Northeast | 366 | 4.3 | 5,665 | 5.7 | 427 | 4.3 | 446 | 4.5 |
| Midwest | 1,885 | 22.2 | 25,310 | 25.3 | 2,271 | 22.7 | 2,239 | 22.4 |
| South | 5,294 | 62.3 | 55,784 | 55.7 | 6,149 | 61.5 | 6,198 | 62.0 |
| West | 954 | 11.2 | 13,402 | 13.4 | 1,148 | 11.5 | 1,112 | 11.1 |
| *Cohort Entry (Year)* |  |  |  |  |  |  |  |  |
| 2010 | 2,476 | 29.1 | 34,199 | 34.1 | 2,484 | 24.9 | 2,484 | 24.9 |
| 2011 | 2,136 | 25.1 | 22,702 | 22.7 | 2,578 | 25.8 | 2,578 | 25.8 |
| 2012 | 2,087 | 24.6 | 22,492 | 22.5 | 2,641 | 26.4 | 2,641 | 26.4 |
| 2013 | 1,800 | 21.2 | 20,768 | 20.7 | 2,292 | 22.9 | 2,292 | 22.9 |

| **Table S1a. Baseline Descriptive Characteristics for All Initiators, Pre- and Post-Matching^1^, Acute Pancreatitis Cohort Optum Research Database Initiation period: February 1, 2010 - December 31, 2013**  **Follow-Up Through March 31, 2014** | | | | | | | | | | | | | | |
| --- | --- | --- | --- | --- | --- | --- | --- | --- | --- | --- | --- | --- | --- | --- |
| **Description** | **First Initiation at Cohort Entry** | | | | | | | **All Matched Initiators  (Initial and Subsequent Initiations)^2^** | | | | | | |
|  | **Liraglutide N=8,499** | | | | **All Comparators N=100,161** | | | **Liraglutide N=9,995** | | | **All Comparators N=9,995** | | | |
|  | **N** | | **%** | | **N** | **%** | | **N** | | **%** | **N** | | | **%** |
| **Healthcare Utilization** |  | |  | |  |  | | |  |  | |  | |  |
| *Doctor cost ($) (median, IQR)* | 745 | | 361-1,718 | | 549 | 245-1,392 | | | 750 | 365-1,736 | | 741 | | 364-1,789 |
| *Medication cost ($) (median, IQR)* | 1,998 | | 995-3,478 | | 513 | 158-1,432 | | | 1,915 | 980-3,334 | | 1,905 | | 965-3,094 |
| *Total cost ($) (median, IQR)* | 3,675 | | 2,052-6,509 | | 1,835 | 762-4,305 | | | 3,578 | 2,024-6,394 | | 3,487 | | 1,980 - 6,414 |
| *No. 3-digit Diagnosis codes* |  |  | |  | | |  | |  |  |  | |  | |
| 0-5 | 1,906 | 22.4 | | 30,821 | | | 30.8 | | 2,233 | 22.3 | 2,246 | | 22.5 | |
| 6-8 | 2,064 | 24.3 | | 24,146 | | | 24.1 | | 2,414 | 24.2 | 2,408 | | 24.1 | |
| 9-13 | 2,308 | 27.2 | | 24,548 | | | 24.5 | | 2,748 | 27.5 | 2,746 | | 27.5 | |
| 14+ | 2,221 | 26.1 | | 20,646 | | | 20.6 | | 2,600 | 26.0 | 2,595 | | 26.0 | |
| *Number of Drugs* |  |  | |  | | |  | |  |  |  | |  | |
| 0-4 | 830 | 9.8 | | 30,998 | | | 30.9 | | 943 | 9.4 | 1,004 | | 10.0 | |
| 5-7 | 1,990 | 23.4 | | 31,420 | | | 31.4 | | 2,380 | 23.8 | 2,348 | | 23.5 | |
| 8-10 | 2,337 | 27.5 | | 19,895 | | | 19.9 | | 2,791 | 27.9 | 2,786 | | 27.9 | |
| 11+ | 3,342 | 39.3 | | 17,848 | | | 17.8 | | 3,881 | 38.8 | 3,857 | | 38.6 | |
| *Number of Visits* |  |  | |  | | |  | |  |  |  | |  | |
| 0-1 | 893 | 10.5 | | 22,170 | | | 22.1 | | 1,035 | 10.4 | 1,027 | | 10.3 | |
| 2-3 | 2,872 | 33.8 | | 36,406 | | | 36.3 | | 3,372 | 33.7 | 3,392 | | 33.9 | |
| 4-5 | 2,122 | 25.0 | | 20,283 | | | 20.3 | | 2,497 | 25.0 | 2,477 | | 24.8 | |
| 6+ | 2,612 | 30.7 | | 21,302 | | | 21.3 | | 3,091 | 30.9 | 3,099 | | 31.0 | |
| *Any ER visit* | 1,557 | 18.3 | | 19,197 | | | 19.2 | | 1,870 | 18.7 | 1,908 | | 19.1 | |
| *Any Hospitalization* | 390 | 4.6 | | 6,800 | | | 6.8 | | 476 | 4.8 | 469 | | 4.7 | |
| *Lab Tests (median, IQR)* | 8 | 4.0-14.0 | | 6 | | | 3.0-12.0 | | 8 | 4.0-14.0 | 8 | | 4.0-14.0 | |
| *Number of Procedures (median, IQR)* | 1 | 0.0-2.0 | | 1 | | | 0.0-2.0 | | 1 | 0.0-2.0 | 1 | | 0.0-2.0 | |
| *Enrollment Days (median, IQR)* | 456 | 307-713 | | 424 | | | 285- 704 | | 517 | 334 - 816 | 543 | | 337 - 858 | |

**Table S1a. Baseline Descriptive Characteristics for All Initiators, Pre- and Post-Matching^1^, Acute Pancreatitis Cohort
Optum Research Database
Initiation period: February 1, 2010 - December 31, 2013**

**Follow-Up Through March 31, 2014**

| **Description** | | **First Initiation at Cohort Entry** | | | | | | | | | **All Matched Initiators  (Initial and Subsequent Initiations)^2^** | | | | | | |
| --- | --- | --- | --- | --- | --- | --- | --- | --- | --- | --- | --- | --- | --- | --- | --- | --- | --- |
|  |  | **Liraglutide N=8,499** | | | | **All Comparators N=100,161** | | | | | **Liraglutide N=9,995** | | | **All Comparators N=9,995** | | | |
|  |  | **N** | | **%** | | **N** | | **%** | | | **N** | | **%** | **N** | | | **%** |
| **Baseline Conditions** | |  |  | |  | | | |  | | |  |  |  | |  | |
| *Overweight* | | 1,670 | 19.6 | | 12,544 | | | | 12.5 | | | 1,941 | 19.4 | 1,912 | | 19.1 | |
| *DCSI Score^3^* | |  |  | |  | | | |  | | |  |  |  | |  | |
| 0 | | 6,072 | 71.4 | | 79,385 | | | | 79.3 | | | 7,159 | 71.6 | 7,115 | | 71.2 | |
| 1 | | 1,320 | 15.5 | | 10,598 | | | | 10.6 | | | 1,526 | 15.3 | 1,571 | | 15.7 | |
| 2 | | 696 | 8.2 | | 6,532 | | | | 6.5 | | | 820 | 8.2 | 807 | | 8.1 | |
| 3+ | | 411 | 4.8 | | 3,646 | | | | 3.6 | | | 490 | 4.9 | 502 | | 5.0 | |
| **History of Baseline Diseases/Procedures** | |  |  | |  | | | |  | | |  |  |  | |  | |
| *Type 1 Diagnosis* | | 152 | 1.8 | | 813 | | | | 0.8 | | | 152 | 1.5 | 125 | | 1.3 | |
| *Type 2 Diagnosis* | | 7,231 | 85.1 | | 70,319 | | | | 70.2 | | | 8,581 | 85.9 | 8,604 | | 86.1 | |
| *No Diabetes Diagnosis* | | 1,116 | 13.1 | | 29,029 | | | | 29.0 | | | 1,262 | 12.6 | 1,266 | | 12.7 | |
| *Pancreatic Disease* | | 1 | 0.0 | | 31 | | | | 0.0 | | | 2 | 0.0 | 4 | | 0.0 | |
| *Abdominal Pain* | | 714 | 8.4 | | 8,746 | | | | 8.7 | | | 872 | 8.7 | 837 | | 8.4 | |
| *Cholelithiasis* | | 30 | 0.4 | | 513 | | | | 0.5 | | | 41 | 0.4 | 46 | | 0.5 | |
| *Status Post Cholecystectomy* | 29 | | 0.3 | | | | 335 | | 0.3 | 40 | | | 0.4 | | 33 | 0.3 | |
| *Abdominal Ultrasound* | 267 | | 3.1 | | | | 2,948 | | 2.9 | 324 | | | 3.2 | | 312 | 3.1 | |
| *Diabetes-related Nephropathy* | 358 | | 4.2 | | | | 1,861 | | 1.9 | 397 | | | 4.0 | | 361 | 3.6 | |
| *Diabetes-related Neuropathy* | 691 | | 8.1 | | | | 3,594 | | 3.6 | 766 | | | 7.7 | | 764 | 7.6 | |
| *Diabetes-related Retinopathy* | 273 | | 3.2 | | | | 1,600 | | 1.6 | 307 | | | 3.1 | | 289 | 2.9 | |
| *Essential Hypertension* | 5,343 | | 62.9 | | | | 52,742 | | 52.7 | 6,349 | | | 63.5 | | 6,312 | 63.2 | |
| *Hyperlipidemia* | 5,739 | | 67.5 | | | | 52,561 | | 52.5 | 6,758 | | | 67.6 | | 6,762 | 67.7 | |
|  | | | | | | | | | | | | | | |  |  | |

**Table S1a. Baseline Descriptive Characteristics for All Initiators, Pre- and Post-Matching^1^, Acute Pancreatitis Cohort
Optum Research Database
Initiation period: February 1, 2010 - December 31, 2013**

**Follow-Up Through March 31, 2014**

| **Description** | **First Initiation at Cohort Entry** | | | | | | **All Matched Initiators  (Initial and Subsequent Initiations)^2^** | | | | | |  |
| --- | --- | --- | --- | --- | --- | --- | --- | --- | --- | --- | --- | --- | --- |
|  | **Liraglutide N=8,499** | | | **All Comparators N=100,161** | | | **Liraglutide N=9,995** | | | **All Comparators N=9,995** | | |  |
|  | **N** | | **%** | **N** | | **%** | **N** | | **%** | **N** | | **%** |  |
| **Baseline Health Behaviors** |  |  | |  |  | |  |  | |  |  | |  |
| *Smoking (positive)* | 227 | 2.7 | | 4,110 | 4.1 | | 292 | 2.9 | | 261 | 2.6 | |  |
| *Alcohol (positive)* | 24 | 0.3 | | 502 | 0.5 | | 30 | 0.3 | | 30 | 0.3 | |  |
| **Baseline Drug Use** |  |  | |  |  | |  |  | |  |  | |  |
| *Fibrates* | 847 | 10.0 | | 7,336 | 7.3 | | 1,013 | 10.1 | | 1,009 | 10.1 | |  |
| *Cholesterol-lowering Drugs* | 4,745 | 55.8 | | 43,218 | 43.1 | | 5,573 | 55.8 | | 5,561 | 55.6 | |  |
| *Insulin Use* | 2,309 | 27.2 | | 8,704 | 8.7 | | 2,533 | 25.3 | | 2,417 | 24.2 | |  |
| *Number of Unique Antidiabetic Drugs^4^* |  |  | |  |  | |  |  | |  |  | |  |
| 0 | 2,342 | 27.6 | | 65,842 | 65.7 | | 2,517 | 25.2 | | 2,529 | 25.3 | |  |
| 1 | 2,950 | 34.7 | | 23,984 | 23.9 | | 3,537 | 35.4 | | 3,593 | 35.9 | |  |
| 2+ | 3,207 | 37.7 | | 10,335 | 10.3 | | 3,941 | 39.4 | | 3,873 | 38.7 | |  |
| ¹Patients were allowed to initiate multiple times during the study period and to match only once into each drug cohort pair.  ^2^Patients who do not match based on their first initiated drug were eligible to be matched based on subsequent drug initiations. | | | | | | | | | | | | | |
| ^3^Diabetes Complications Severity Index  ^4^Not including insulin. | | | | | | | | | | | | | |

| **Table S1b. Baseline Descriptive Characteristics for All Initiators, Pre and Post Matching^1^, Pancreatic Cancer Cohort  Optum Research Database Initiation Period: February 1, 2010 - November 30, 2014 Follow-Up Through December 31, 2014** | | | | | | | | |
| --- | --- | --- | --- | --- | --- | --- | --- | --- |
| **Description** | **First Initiation at Cohort Entry** | | | | **All Matched Initiators  (Initial and Subsequent Initiations)^2^** | | | |
|  | **Liraglutide N=27,283** | | **All Comparators N=362,539** | | **Liraglutide N=35,163** | | **All Comparators N=35,163** | |
|  | **N** | **%** | **N** | **%** | **N** | **%** | **N** | **%** |
| **Demographic** |  |  |  |  |  |  |  |  |
| *Age (mean (median), IQR)* | 52(53) | 45.0-59.0 | 52(53) | 44.0-61.0 | 52(53) | 46.0-60.0 | 52(53) | 46.0-59.0 |
| 18-39 | 3,181 | 11.7 | 60,908 | 16.8 | 3,970 | 11.3 | 3,974 | 11.3 |
| 40-49 | 6,898 | 25.3 | 78,217 | 21.6 | 8,848 | 25.2 | 8,983 | 25.5 |
| 50-59 | 10,549 | 38.7 | 120,024 | 33.1 | 13,516 | 38.4 | 13,540 | 38.5 |
| 60-64 | 4,594 | 16.8 | 58,895 | 16.2 | 5,999 | 17.1 | 5,950 | 16.9 |
| 65+ | 2,061 | 7.6 | 44,495 | 12.3 | 2,830 | 8.0 | 2,716 | 7.7 |
| *Gender* |  |  |  |  |  |  |  |  |
| Female | 14,644 | 53.7 | 178,007 | 49.1 | 18,712 | 53.2 | 18,699 | 53.2 |
| Male | 12,639 | 46.3 | 184,532 | 50.9 | 16,451 | 46.8 | 16,464 | 46.8 |
| *Race* |  |  |  |  |  |  |  |  |
| Black | 3,283 | 12.0 | 50,420 | 13.9 | 4,282 | 12.2 | 4,971 | 14.1 |
| *Region* |  |  |  |  |  |  |  |  |
| Northeast | 1,865 | 6.8 | 26,233 | 7.2 | 2,393 | 6.8 | 2,307 | 6.6 |
| Midwest | 5,996 | 22.0 | 96,389 | 26.6 | 7,980 | 22.7 | 7,941 | 22.6 |
| South | 16,165 | 59.2 | 187,164 | 51.6 | 20,542 | 58.4 | 20,647 | 58.7 |
| West | 3,257 | 11.9 | 52,753 | 14.6 | 4,248 | 12.1 | 4,268 | 12.1 |
| *Cohort Entry (Year)* |  |  |  |  |  |  |  |  |
| 2010 | 7,334 | 26.9 | 111,954 | 30.9 | 7,911 | 22.5 | 7,911 | 22.5 |
| 2011 | 6,211 | 22.8 | 70,601 | 19.5 | 8,059 | 22.9 | 8,059 | 22.9 |
| 2012 | 5,495 | 20.1 | 64,934 | 17.9 | 7,809 | 22.2 | 7,809 | 22.2 |
| 2013 | 4,705 | 17.2 | 58,766 | 16.2 | 6,415 | 18.2 | 6,415 | 18.2 |
| 2014 | 3,538 | 13.0 | 56,284 | 15.5 | 4,969 | 14.1 | 4,969 | 14.1 |
| **Healthcare Utilization** |  |  |  |  |  |  |  |  |
| *Doctor Cost ($) (median, IQR)* | 716 | 340-1,644 | 544 | 239-1,389 | 818 | 391-1,904 | 830 | 395-1,966 |
| *Medication Cost ($) (median, IQR)* | 2,143 | 1,057-3,727 | 551 | 166-1,541 | 2,104 | 1,061-3,655 | 1,926 | 1,014-3,223 |

| **Table S1b. Baseline Descriptive Characteristics for All Initiators, Pre and Post Matching^1^, Pancreatic Cancer Cohort  Optum Research Database Initiation Period: February 1, 2010 - November 30, 2014 Follow-Up Through December 31, 2014** | | | | | | | | | | | | | |
| --- | --- | --- | --- | --- | --- | --- | --- | --- | --- | --- | --- | --- | --- |
| **Description** | **First Initiation at Cohort Entry** | | | | | **All Matched Initiators  (Initial and Subsequent Initiations)^2^** | | | | | | | |
|  | **Liraglutide N=27,283** | | **All Comparators N=362,539** | | | **Liraglutide N=35,163** | | | | **All Comparators N=35,163** | | | |
|  | **N** | **%** | **N** | | **%** | **N** | | | **%** | **N** | | | **%** |
| *Total Cost ($) (median, IQR)* | 3,947 | 2,183-7,050 | 1,991 | | 832-4,641 | 3,920 | 2,185-7,011 | | | 3,705 | 2,166-6,843 | | |
| *No. 3-digit Diagnosis codes* |  |  |  | |  |  |  | | |  |  | | |
| 0-5 | 5,739 | 21.0 | 103,314 | | 28.5 | 7,293 | 20.7 | | | 7,389 | 21.0 | | |
| 6-8 | 6,281 | 23.0 | 84,663 | | 23.4 | 8,172 | 23.2 | | | 8,131 | 23.1 | | |
| 9-13 | 7,702 | 28.2 | 91,206 | | 25.2 | 9,852 | 28.0 | | | 9,835 | 28.0 | | |
| 14+ | 7,561 | 27.7 | 83,356 | | 23.0 | 9,846 | 28.0 | | | 9,808 | 27.9 | | |
| *Number of Drugs* |  |  |  | |  |  |  | | |  |  | | |
| 0-4 | 2,583 | 9.5 | 106,877 | | 29.5 | 3,099 | 8.8 | | | 3,231 | 9.2 | | |
| 5-7 | 6,057 | 22.2 | 111,263 | | 30.7 | 7,822 | 22.2 | | | 7,862 | 22.4 | | |
| 8-10 | 7,269 | 26.6 | 73,182 | | 20.2 | 9,572 | 27.2 | | | 9,502 | 27.0 | | |
| 11+ | 11,374 | 41.7 | 71,217 | | 19.6 | 14,670 | 41.7 | | | 14,568 | 41.4 | | |
| *Number of Visits* |  |  |  | |  |  |  | | |  |  | | |
| 0-1 | 2,731 | 10.0 | 76,122 | | 21.0 | 3,384 | 9.6 | | | 3,433 | 9.8 | | |
| 2-3 | 8,773 | 32.2 | 128,593 | | 35.5 | 11,353 | 32.3 | | | 11,347 | 32.3 | | |
| 4-5 | 6,937 | 25.4 | 73,368 | | 20.2 | 8,901 | 25.3 | | | 8,872 | 25.2 | | |
| 6+ | 8,842 | 32.4 | 84,456 | | 23.3 | 11,525 | 32.8 | | | 11,511 | 32.7 | | |
| *Any ER visit* | 4,963 | 18.2 | 71,340 | | 19.7 | 6,484 | 18.4 | | | 6,450 | 18.3 | | |
| *Any Hospitalization* | 1,252 | 4.6 | 26,481 | | 7.3 | 1,716 | 4.9 | | | 1,664 | 4.7 | | |
| *Lab Tests (median, IQR)* | 8 | 4.0 - 14.0 | 6 | | 3.0-12.0 | 8 | 4.0-14.0 | | | 8 | 4.0-14.0 | | |
| *Number of Procedures (median, IQR)* | 1 | 0.0 - 3.0 | 1 | | 0.0-2.0 | 1 | 0.0-3.0 | | | 1 | 0.0-3.0 | | |
| *Enrollment Days (median, IQR)* | 453 | 310.0 - 741.0 | 332 | | 251.0-439.0 | 609 | 373.0-993.0 | | | 644 | 389.0-1,034.0 | | |
| **Baseline Conditions** |  |  |  | |  |  |  | | |  |  | | |
| *Overweight* | 5,786 | 21.2 | 48,929 | | 13.5 | 7,478 | 21.3 | | | 7,494 | 21.3 | | |
| *DCSI Score^3^* |  |  |  | |  |  |  | | |  |  | | |
| 0 | 19,413 | 71.2 | 282,762 | | 78.0 | 24,965 | 71.0 | | | 24,593 | 69.9 | | |
| 1 | 4,207 | 15.4 | 38,295 | | 10.6 | 5,381 | 15.3 | | | 5,624 | 16.0 | | |
| 2 | 2,237 | 8.2 | 25,788 | | 7.1 | 2,953 | 8.4 | | | 3,048 | 8.7 | | |
| 3+ | 1,426 | 5.2 | 15,694 | | 4.3 | 1,864 | 5.3 | | | 1,898 | 5.4 | | |
| **Table S1b. Baseline Descriptive Characteristics for All Initiators, Pre and Post Matching^1^, Pancreatic Cancer Cohort  Optum Research Database Initiation Period: February 1, 2010 - November 30, 2014 Follow-Up Through December 31, 2014** | | | | | | | | | | | | | |
| **Description** | **First Initiation at Cohort Entry** | | | | | **All Matched Initiators  (Initial and Subsequent Initiations)^2^** | | | | | | | |
|  | **Liraglutide N=27,283** | | **All Comparators N=362,539** | | | **Liraglutide N=35,163** | | | | **All Comparators N=35,163** | | | |
|  | **N** | **%** | **N** | | **%** | **N** | | | **%** | **N** | | | **%** |
| ***History of Baseline Diseases/Procedures*** |  |  |  | |  |  |  | | |  |  | | |
| *Type 2 Diabetes* | 23,239 | 85.2 | 254,223 | | 70.1 | 30,505 | 86.8 | | | 30,671 | 87.2 | | |
| *Type 1 Diabetes* | 486 | 1.8 | 2,686 | | 0.7 | 529 | 1.5 | | | 439 | 1.2 | | |
| *No Diabetes Diagnosis* | 3,558 | 13.0 | 105,623 | | 29.1 | 4,129 | 11.7 | | | 4,052 | 11.5 | | |
| *Benign Thyroid Disease* | 3,702 | 13.6 | 32,066 | 8.8 | | 4,629 | | 13.2 | | 4,579 | | 13.0 | |
| *Goiters/Nodules* | 960 | 3.5 | 6,901 | 1.9 | | 1,199 | | 3.4 | | 1,180 | | 3.4 | |
| *Pancreatic Disease* | 62 | 0.2 | 1,658 | 0.5 | | 79 | | 0.2 | | 131 | | 0.4 | |
| *Acute Pancreatitis* | 46 | 0.2 | 1,291 | 0.4 | | 57 | | 0.2 | | 102 | | 0.3 | |
| *Chronic Pancreatitis* | 15 | 0.1 | 401 | 0.1 | | 21 | | 0.1 | | 34 | | 0.1 | |
| *Diabetes-related Nephropathy* | 1,162 | 4.3 | 7,570 | 2.1 | | 1,510 | | 4.3 | | 1,421 | | 4.0 | |
| *Diabetes-related Neuropathy* | 2,177 | 8.0 | 13,634 | 3.8 | | 2,772 | | 7.9 | | 2,762 | | 7.9 | |
| *Diabetes-related Retinopathy* | 865 | 3.2 | 5,672 | 1.6 | | 1,084 | | 3.1 | | 1,065 | | 3.0 | |
| *Acute MI* | 230 | 0.8 | 4,298 | 1.2 | | 323 | | 0.9 | | 316 | | 0.9 | |
| *Stroke* | 364 | 1.3 | 6,041 | 1.7 | | 486 | | 1.4 | | 561 | | 1.6 | |
| *Acute Heart Failure* | 439 | 1.6 | 7,122 | 2.0 | | 591 | | 1.7 | | 626 | | 1.8 | |
| *Acute Renal Failure* | 444 | 1.6 | 7,545 | 2.1 | | 612 | | 1.7 | | 617 | | 1.8 | |
| *Essential Hypertension* | 17,416 | 63.8 | 192,675 | 53.1 | | 22,721 | | 64.6 | | 22,820 | | 64.9 | |
| *Hyperlipidemia* | 18,336 | 67.2 | 189,873 | 52.4 | | 23,858 | | 67.8 | | 23,698 | | 67.4 | |
| **Baseline Health Behaviors** |  |  |  |  | |  | |  | |  | |  | |
| *Smoking (positive)* | 781 | 2.9 | 14,801 | 4.1 | | 1,064 | | 3.0 | | 1,107 | | 3.1 | |
| *Alcohol (positive)* | 63 | 0.2 | 2,049 | 0.6 | | 82 | | 0.2 | | 118 | | 0.3 | |

| **Table S1b. Baseline Descriptive Characteristics for All Initiators, Pre and Post Matching^1^, Pancreatic Cancer Cohort  Optum Research Database Initiation Period: February 1, 2010 - November 30, 2014 Follow-Up Through December 31, 2014** | | | | | | | | | | | | |
| --- | --- | --- | --- | --- | --- | --- | --- | --- | --- | --- | --- | --- |
| **Description** | **First Initiation at Cohort Entry** | | | | | **All Matched Initiators  (Initial and Subsequent Initiations)^2^** | | | | | | |
|  | **Liraglutide N=27,283** | | **All Comparators N=362,539** | | | **Liraglutide N=35,163** | | | **All Comparators N=35,163** | | | |
|  | **N** | **%** | **N** | | **%** | **N** | | **%** | **N** | | **%** | |
| **Baseline Drug Dispensings** |  |  |  |  | |  |  | |  |  | | |
| *Fibrates* | 2,617 | 9.6 | 25,977 | 7.2 | | 3,492 | 9.9 | | 3,544 | 10.1 | | |
| *Cholesterol-lowering Drugs* | 15,273 | 56.0 | 160,058 | 44.1 | | 19,825 | 56.4 | | 19,595 | 55.7 | | |
| *Insulin Use* | 7,762 | 28.4 | 32,370 | 8.9 | | 9,387 | 26.7 | | 9,266 | 26.4 | | |
| *Number of Unique Antidiabetic Drugs^4^* |  |  |  |  | |  |  | |  |  | | |
| 0 | 7,747 | 28.4 | 240,394 | 66.3 | | 8,682 | 24.7 | | 8,552 | 24.3 | | |
| 1 | 9,676 | 35.5 | 86,422 | 23.8 | | 12,677 | 36.1 | | 12,899 | 36.7 | | |
| 2+ | 9,860 | 36.1 | 35,723 | 9.9 | | 13,804 | 39.3 | | 13,712 | 39.0 | | |
| ¹Patients were allowed to initiate multiple times during the study period and to match only once into each drug cohort pair.  ^2^Patients who do not match based on their first initiated drug were eligible to be matched based on subsequent drug initiations. | | | | | | | | | | | |  |
| ^3^Diabetes Complications Severity Index  ^4^Not including insulin. | | | | | | | | | | | |  |

**Supplemental Appendix 2. Acute Pancreatitis Case Adjudication**

There were 320 AP cases identified from primary inpatient diagnoses (Table S1a). These are cases where the diagnosis code for AP was entered in the first position on the hospital claims form, a position that is thought to reflect a primary reason for the hospitalization. An additional 88 initiators had an inpatient diagnosis that was not reported in the primary position (non-primary inpatient). Thirty potential cases were randomly sampled from this group to quantify the extent to which cases were missed by requiring the primary position. Records were received for over 85% of both groups. Sixty percent of the primary inpatient cases were confirmed as definite or probable AP cases compared with 23% of those with non-primary diagnoses. A total of 180 AP cases were confirmed by the adjudicators. Fifteen of these were dropped as noted in Table S-1. These exclusions left 165 AP cases for the analysis. Follow-up was censored at the claims-date of AP.

**Table S2. Summary of Chart Adjudication for Acute Pancreatitis**

|  | **Diagnosis code in Primary Position on Inpatient Claim** | | **Diagnosis code in Non-Primary Position on Inpatient Claim⁴** | |
| --- | --- | --- | --- | --- |
|  | **N** | **%** | **N** | **%** |
| Records requested | 320 |  | 30 |  |
| Records received | 288 | 90.0 | 26 | 86.7 |
| Confirmed | 174 | 60.4 | 6 | 23.1 |
| **Total Cases (Primary & Non-Primary)** | 180 |  |  |  |
| Status changed¹ | 4 |  |  |  |
| Dropped: prior exclusions² | 9 |  |  |  |
| Dropped: chart information³ | 2 |  |  |  |
| **Final case count** | **165** |  |  |  |

^1^Four charts could not be used because the patients’ health plan statuses changed to a plan that does not allow linkage to medical records.

_2_Additional data identified baseline exclusions were present.

_3_Chart information supported prior pancreatic cancer.

⁴Potential cases that had a diagnosis code entered in a secondary position on the hospital claims form. Thirty were randomly selected to determine the confirmation rate for non-primary inpatient codes.

**Supplemental Appendix 3. Time from Initiation to Outcome**

**Table S3a. Time to Acute Pancreatitis¹ Diagnosis in Days (All First Initiations and Matched^2^ Liraglutide/Study Drug Pairs)**

|  | | | | **Matched Pairs (First and Subsequent Initiations)** | | | | | |
| --- | --- | --- | --- | --- | --- | --- | --- | --- | --- |
|  | **All First Initiations** | | | **Liraglutide** | | | **Comparators** | | |
| **Study Drug** | **Case N** | **Median** | **IQR** | **Case N** | **Median** | **IQR** | **Case N** | **Median** | **IQR** |
| Liraglutide and Comparators | 165 | 308 | 128 - 583 |  |  |  |  |  |  |
| Liraglutide | 21 | 421 | 248 - 611 |  |  |  |  |  |  |
| All Comparators | 144 | 286 | 127 - 565 | 21 | 444 | 121 - 611 | 11 | 250 | 112 - 381 |
| All Comparators (excluding Exenatide) | 140 | 284 | 127 - 581 | 24 | 330 | 103 - 597 | 17 | 192 | 128 - 337 |
| All Comparators (excluding Exenatide and DPP-4i) | 123 | 281 | 128 - 566 | 20 | 378 | 103 - 597 | 8 | 249 | 147 - 547 |
| Exenatide | 4 | 355 | 203 - 426 | 8 | 639 | 255 - 909 | 4 | 203 | 63 - 379 |
| DPP-4i  (Saxagliptin/Sitagliptin/Linagliptin) | 17 | 345 | 112 - 595 | 16 | 378 | 119 - 543 | 13 | 139 | 65 - 345 |
| Metformin | 74 | 286 | 177 - 689 | 17 | 421 | 89 - 583 | 10 | 249 | 128 - 398 |
| Sulfonylureas (Glyburide/Glipizide/Glimepiride) | 40 | 241 | 112 - 383 | 15 | 421 | 248 - 583 | 13 | 314 | 125 - 501 |
| Pioglitazone | 9 | 430 | 79 - 563 | 8 | 487 | 373 - 639 | 2 | 304 | 50 - 557 |

IQR; Interquartile Range

¹Time from day after index date to acute pancreatitis diagnosis date based on adjudication.

2Patients were allowed to initiate multiple times during the study period and to match only once into each drug cohort pair.

**Table S3b. Time to Pancreatic Cancer¹ Diagnosis in Days (All First Initiations and Matched^2^ Liraglutide/Study Drug Pairs)**

| **Study Drug** | **All First Initiations** | | | **Matched Pairs (First and Subsequent Initiations)** | | | | | |
| --- | --- | --- | --- | --- | --- | --- | --- | --- | --- |
|  |  |  |  | **Liraglutide** | | | **Comparators** | | |
|  | **Cases** | **Median** | **IQR** | **Cases** | **Median** | **IQR** | **Cases** | **Median** | **IQR** |
| Liraglutide And Comparators | 240 | 327 | 162 - 640 |  |  |  |  |  |  |
| Liraglutide | 10 | 369 | 226 - 1,099 |  |  |  |  |  |  |
| All Comparators | 230 | 318 | 161 - 627 | 13 | 333 | 158 - 594 | 19 | 317 | 168 - 627 |
| All Comparator Drugs Except Exenatide | 227 | 314 | 161 - 653 | 15 | 333 | 108 - 594 | 19 | 275 | 164 - 511 |
| All Comparator Drugs Except Exenatide And DPP-4i | 199 | 314 | 151 - 665 | 12 | 192 | 94 - 464 | 19 | 511 | 244 - 785 |
| Exenatide | 3 | 491 | 36 - 498 | 8 | 369 | 144 - 573 | 5 | 168 | 36 - 491 |
| DPP-4i (Saxagliptin/Sitagliptin/Linagliptin) | 28 | 335 | 169 - 611 | 10 | 246 | 108 - 551 | 8 | 314 | 164 - 737 |
| Metformin | 102 | 339 | 163 - 715 | 9 | 226 | 108 - 594 | 12 | 553 | 166 - 825 |
| Sulfonylureas (Glyburide/Glipizide/Glimepiride) | 77 | 243 | 126 - 447 | 11 | 362 | 108 - 1,099 | 24 | 339 | 177 - 525 |
| Pioglitazone | 20 | 857 | 306 - 1,120 | 8 | 573 | 133 - 1,104 | 10 | 417 | 233 - 909 |

IQR, interquartile range

^1^Time from day after index date to first date of ICD-9 code 157 for all cases.

2Patients were allowed to initiate multiple times during the study period and to match only once into each drug cohort pair.
